# Complications and Risk Factors of Neurogenic Bladder: A Delphi Consensus

**DOI:** 10.1016/j.euros.2026.02.005

**Published:** 2026-02-21

**Authors:** Nicolas Turmel, Camille Chesnel, Pierre Denys, Xavier Game, Gérard Amarenco, Claire Hentzen

**Affiliations:** a Public Assistance Hospitals, Paris, France; b GRC-01 Group of Clinical Research in Neuro-Urology, Sorbonne University, Paris, France; c Department of Rehabilitation Medicine and Pediatric Readaptation, Hospital Raymond-Poincare, Garches, France; d Department of Urology Andrology and Renal Transplantation, University Hospital Centre, Toulouse, France; e Sorbonne Paris North university; f Physical Medicine and Rehabilitation Department, Casanova Hospital, Saint Denis; g Neuro Urology department, Pitie Salpetriere Hospital, APHP, Paris

**Keywords:** Neurogenic bladder, Delphi methods, Neurourological risk

## Abstract

**Background and objective:**

Lower urinary tract symptoms secondary to neurogenic bladder impact quality of life and symptom burden significantly. These conditions are also associated with serious complications, including febrile urinary tract infections (UTIs) and impaired renal function. This study aimed to specify and rank the neurogenic bladder risk factors, their consequences, and finally the induced complications observed in neurourology.

**Methods:**

We conducted a Delphi consensus between October 2023 and April 2025. A steering committee performed a comprehensive literature review and drafted initial proposals. A review committee and a multidisciplinary expert panel were formed to evaluate and rate the proposals. The standard Delphi methodology was followed throughout.

**Key findings and limitations:**

After three rounds, 22 proposals were validated. Six proposals addressed the complications (renal failure, febrile UTIs, and autonomic dysreflexia), while 16 focused on risk factors, including disease modality (spinal cord injury and myelomeningocele), urodynamic characteristics (detrusor leak point pressure ≥40 cmH_2_O and low bladder compliance), and treatment-related risk factors (use of indwelling or suprapubic catheters). This consensus allows to specification and prioritization of the risk factors, thus providing clinical elements for the assessment and follow-up of patients with neurogenic bladder. While expert agreement was generally strong, the process highlighted a significant lack of validated criteria in the current literature. Most urodynamic thresholds are derived from small, retrospective studies, and carried out in specific populations. The findings underscore the need for better-defined, evidence-based risk stratification in neurourology.

**Conclusions and clinical implications:**

This consensus validated 22 expert-approved proposals, including six addressing the complications and 16 identifying the risk factors associated with neurogenic bladder.

**Patient summary:**

This expert’s consensus provides a better understanding of upper urinary tract complications for patients with neurogenic bladder. It may help practitioners to better distinguish high-risk patients from low-risk patients and improve their management to avoid potential complications (renal failure, urinary tract infections, and other complications).

## Take Home Message

Patients with neurogenic bladder may experience complications in the upper urinary tract, such as renal failure, febrile urinary tract infection, or autonomic dysreflexia. Special attention should be given to patients with spinal cord injury, spina bifida, or multiple sclerosis.

## 1 Introduction

Management of neurogenic lower urinary tract symptoms has been investigated extensively over the past decades. Multiple guidelines have been published and have evolved in parallel with advances in diagnosis and therapeutics. These interventions improve outcomes such as quality of life and reduce the severity of symptoms, both of which are key elements of patient adherence [1], [2]. However, in a specific population with a high risk of complications, reducing risk factors for the prevention of upper urinary tract damage (UUTD) and urinary tract infections (UTIs) is usually the most important criterion, and some treatments specifically target detrusor overactivity to reduce intravesical pressure, allowing an increase in bladder capacity. Historically, appropriate neurogenic bladder management—such as intermittent catheterization and treating detrusor overactivity—has had a significant impact on life-threatening complications, causing a drastic reduction in the rate of deaths related to urogenital complications over time [3].

Bladder management aims to control (1) high pressure related to detrusor overactivity, (2) low bladder compliance, or (3) detrusor sphincter dyssynergia (DSD). Several thresholds have been proposed, but the most widely used are detrusor leak point pressure (DLPP) >40 cmH_2_O and bladder compliance ≤20 ml/cmH_2_O, derived from the retrospective study conducted by McGuire et al [4] in 1981 involving 42 children with spina bifida. The authors argued that addressing these risk factors is essential for preserving renal function and preventing vesicoureteral reflux (VUR). Since then, several risk and predictive scores have incorporated these thresholds—often with minor modifications—usually in populations other than those with spina bifida, without specific validation. In 1991, Galloway et al [5] developed a predictive score for UUTD in spinal dysraphism, combining clinical and urodynamic variables. A score of >5 defined high-risk patients for hydronephrosis within 5 yr. However, the score was developed exclusively in pediatric populations with spinal dysraphism, probably limiting generalizability. Moreover, hydronephrosis is a consequence of abnormal bladder function, and it does not systematically lead to renal failure in all affected populations.

**Fig. 1:**
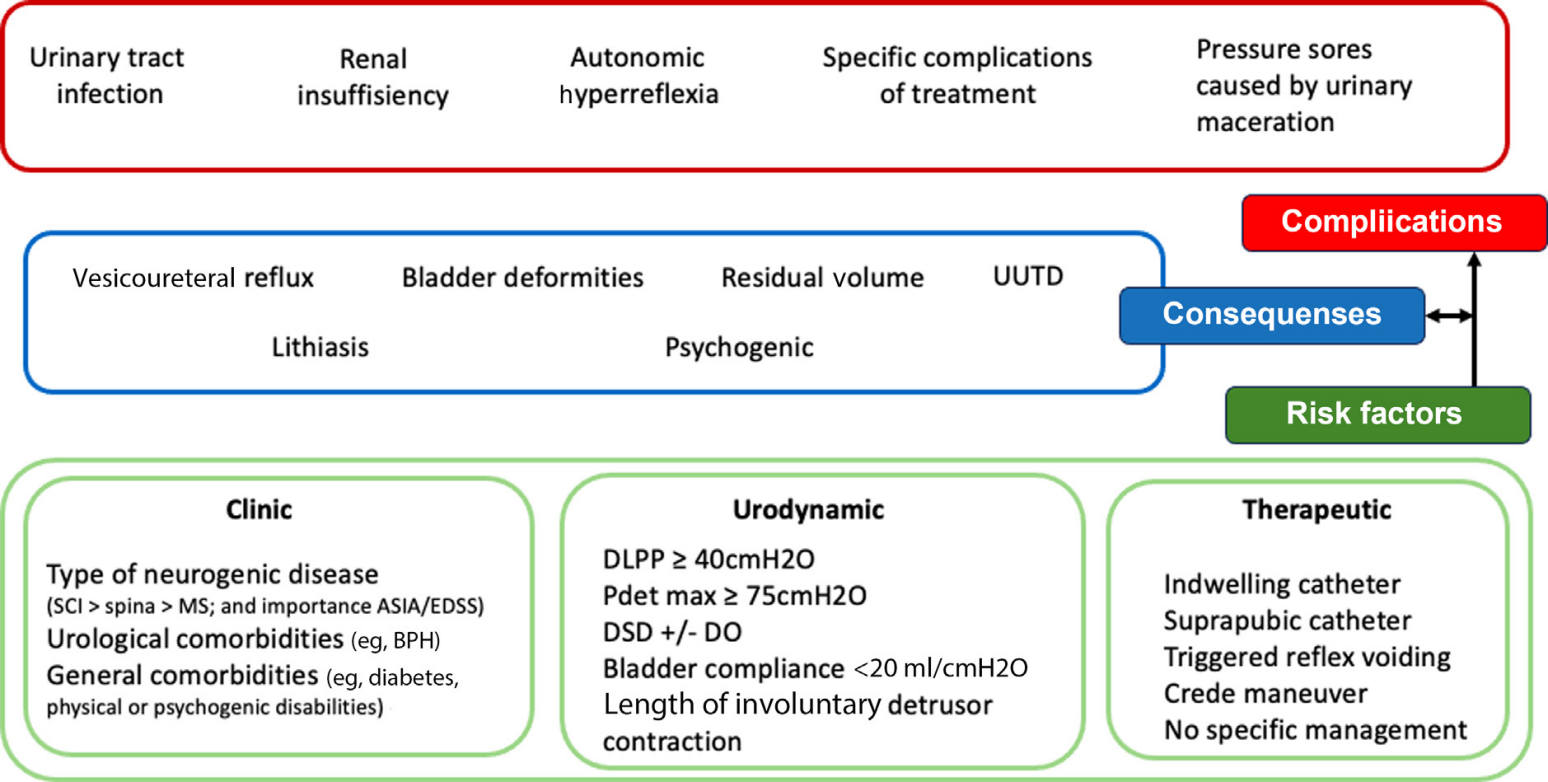
From risk factors to complications in neurourology. ASIA = American Spinal Injury Association; BPH = benign prostatic hyperplasia; DLPP = detrusor leak point pressure; DO = detrusor overactivity; DSD = detrusor sphincter dyssynergia; EDSS = Expanded Disability Status Scale; MS = multiple sclerosis; Pdetmax = maximum detrusor pressure; SCI = spinal cord injury; UUTD = upper urinary tract dilatation.

Research on neurourological complications in spinal dysraphism has been substantial. A recent systematic review by Rague et al [6] highlighted major heterogeneity in the definitions of complications and kidney abnormalities. Of the articles, 15% did not define kidney abnormalities, and although about 65% (177/274) used hydronephrosis as their primary endpoint, only 19.8% (35/177) defined it explicitly. Other publications mixed radiological and biological indicators such as renal scarring (66/274) or creatinine serum-based testing (101/274), and only 9.9% (27/274) used glomerular filtration rate as an endpoint. Risk factors (eg, high detrusor pressure), consequences (eg, bladder deformities and reflux), and confirmed complications (UTIs and renal failure) were conflated frequently (Fig. 1). This variability and a lack of clear definitions limit interpretation and hinder the establishment of validated cutoffs.

This inconsistency likely contributes to the heterogeneity in clinical and urodynamic thresholds. In a systematic review in 2018, Musco et al [7] identified several clinical and urodynamic factors potentially associated with UUTD in neurogenic populations, but were unable to establish robust cutoffs or reliable predictors [8], [9]. The authors emphasized limitations in defining UUTD, including the overuse of VUR and hydronephrosis as endpoints and the differences in neurogenic populations in the evolution and prognosis of neurogenic bladder (myelomeningocele, spinal cord injury [SCI], and multiple sclerosis [MS] among others). Whether the risk of complications is comparable across neurological conditions remains unclear, especially given the influence of individual comorbidities (age, sex, etc.), which are often different between the various neurological diseases. In light of these persistent gaps, evidence-based recommendations cannot yet be formulated, and the current guidance relies largely on expert opinion and consensus statements. This lack of clarity in defining risk factors and complications significantly affects patient management, follow-up, and referral to specialized centers.

To address these challenges, we conducted a nationally funded project entitled HOPE (Hierarchization and Algorithms for Optimizing the Care Pathway for Patients with Neuro-urological Disabilities). This multistep initiative has the following objectives:

1. Define the needs and organization of neurourology units based on their level of expertise and the complexity of cases treated. This will lead to the identification of primary, secondary, and tertiary centers based on expertise, resources, and multidisciplinary capacities, and offer better visibility for referral based on the risk level of complications and the therapeutics required.
2. Standardize the management of specific issues—such as recurrent UTIs or pregnancy in women with neurological disorders.
3. Determine the risk factors that lead to complications, a necessary step to determine the level of expertise required for patient treatment.

Identification of these risk factors through expert consensus is expected to improve patient referral pathways, optimize care according to individual risk profiles (high/moderate/low), and reduce unnecessary health care resource use.

The aim of the study is in line with this third objective, to identify risk factors and complications associated with neurogenic bladder.

## 2 Patients and methods

Between October 2023 and April 2025, a prospective study was conducted to develop expert recommendations using the Delphi method. The study followed the methodological standards reported by Niederberger et al [10] for interdisciplinary Delphi studies and the formal consensus procedures defined by the French High Authority for Health (Haute Autorité de Santé [HAS]) [11].

The steering committee, composed of two independent experts, coordinated the literature review and drafted the initial items for review without consulting external experts during the validation process.

The review committee comprised ten experts selected based on their prior Delphi method experience and academic qualifications. After each round, this committee participated in meetings to assess the clinical relevance of each item, review comments, resolve any duplicates, and reword items for subsequent rounds as needed. They also examined all the comments from the rating committee.

The rating committee comprised 40 experts from diverse medical fields, each with extensive neurourological experience: urology (14 experts), gynecology (one expert), and physical and rehabilitation medicine (25 experts). Experts were selected using a combination of criteria: regular clinical practice involving patients with neurogenic bladder, a minimum of 5 yr of clinical experience, active membership in scientific societies, participation in educational programs related to neurogenic bladder, and academic publications (Table 1). Owing to the limited pool of national experts, five individuals served on both the review and the rating committee. Experts were invited by e-mail and received detailed information about the project, its objectives, and the Delphi procedure. All experts were members or leading members of scientific societies including the French Association of Urology (AFU), the French Interdisciplinary Society of Urodynamics and Pelvi-Perineology (SIFUD-PP), and the French Language Neuro-Urology Research Group (GENULF). Some of these experts were also members of international societies, particularly the International Continence Society and the European Association of Urology.

**Table 1:** Experts’ identification and background

	Degree	Publications (number)	H Index	Specialty	Round participation
Expert 1 (P.D.)	MD, PhD	253	37	PMR	R1/R2/R3
Expert 2 (R.H.)	MD, PhD student	63	10	PMR	R1/R2/R3
Expert 3 (C.C.)	MD	82	8	PMR	R1/R2/R3
Expert 4 (G.A.)	MD, PhD	386	36	PMR	R1/R2/R3
Expert 5 (X.D.)	MD, PhD	285	31	Gynecology	R1/R2/R3
Expert 6 (M.G.)	MD	13	5	PMR	R1/R2/R3
Expert 7 (F.L.B.)	MD	89	12	PMR	R1/R2/R3
Expert 8 (A.G.L.)	MD	27	8	PMR	R1/R2/R3
Expert 9 (B.P.)	MD	15	3	PMR	R1/R2/R3
Expert 10 (M.J.)	MD, PhD	44	11	PMR	R1/R2/R3
Expert 11 (E.V.)	MD	42	10	PMR	R1/R2/R3
Expert 12 (J.G.P.)	MD	41	15	PMR	R1/R2/R3
Expert 13 (J.K.)	MD	86	13	PMR	R1/R2/R3
Expert 14 (S.C.)	MD, PhD	225	26	Urology	R1/R2/R3
Expert 15 (N.H.)	MD	10	4	PMR	R1/R2/R3
Expert 16 (E.V.L.)	MD	77	17	PMR	R1/R2/R3
Expert 17 (J.F.H.)	MD, PhD	171	21	Urology	R1/R2
Expert 18 (P.L.D.)	MD, PhD	19	8	PMR	R1/R2
Expert 19 (M.A.P.V.)	MD, PhD	156	18	Urology	R1
Expert 20 (M.D.S.)	MD, PhD	151	26	PMR	R1
Total (median)		79.5	12.5		

PMR = physical medicine and rehabilitation; R = round.

The steering committee defined a three-round Delphi process, adhering to the French recommendations for consensus guidelines [10]. The first step consisted of a literature review to identify the risk factors and complications in patients with neurogenic bladder, using the following keywords: “neuro-urology,” “urologic complications,” “urinary tract infection,” “neurogenic bladder,” “risk factor,” “detrusor overactivity,” “lower urinary tract symptoms,” “prognosis,” “predictive risk,” “upper urinary tract complication,” “vesicoureteral reflux,” “hydronephrosis,” “renal failure,” “renal impairment,” and “renal insufficiency.” This process produced a list of 51 proposed items for round 1.

In round 1, rating committee experts scored their agreement with each item using a 9-point Likert scale ranging from 1 (strong disagreement) to 9 (strong agreement), with a score of 5 expressing neutrality. Comments were not permitted at this stage. After each round, the steering committee summarized the results for the review committee, which refined, reworded, or consolidated items before the next round. From round 2 onwards, experts were encouraged to comment on items rated below 7.

### 2.1 Data analysis

Rating rules and score interpretation adhered to the French guidelines for formal consensus. For each round, mean values, medians, and standard deviations were presented to the review committee, along with the recommended classification of each item (appropriate for use, uncertain, or inappropriate for use). Results were also communicated to the rating committee before each subsequent round.

**Fig. 2:**
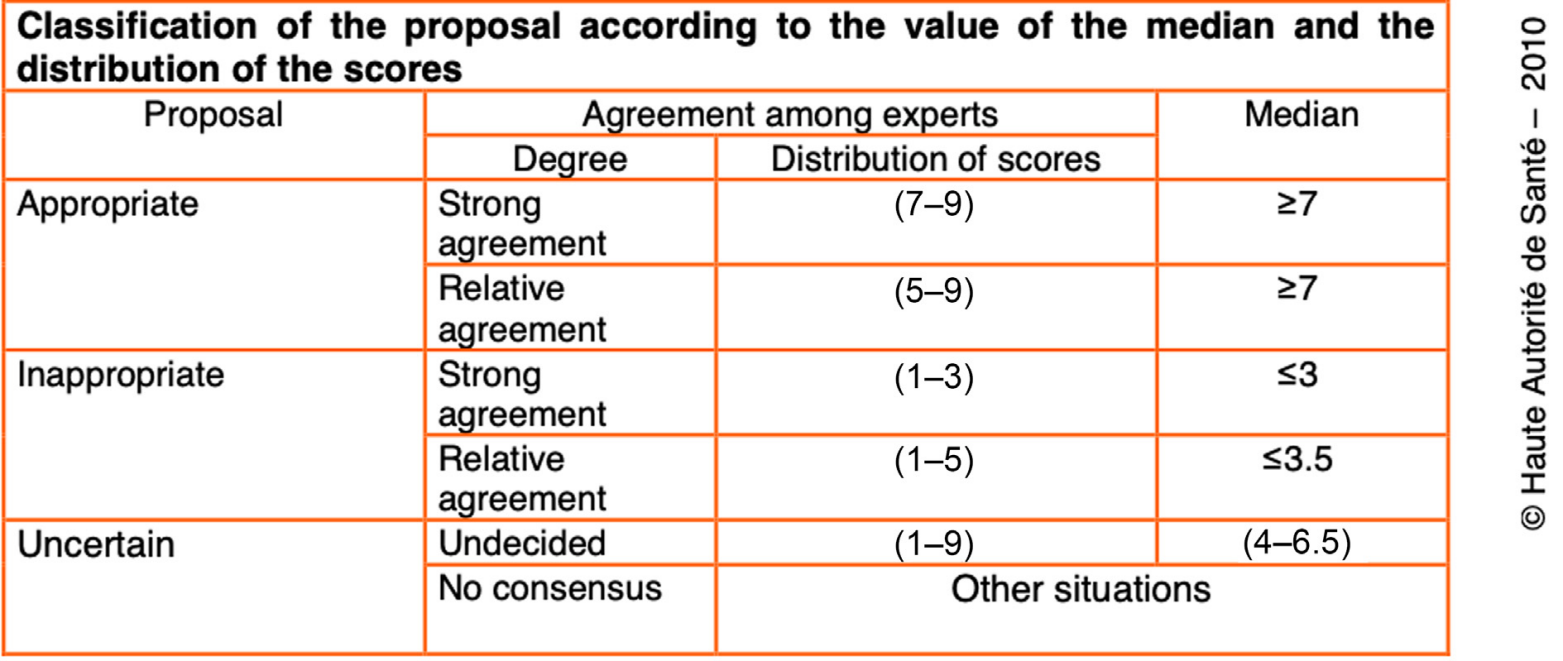
Classification of the proposal (HAS 2010). HAS = Haute Autorité de Santé (French High Authority for Health).

In round 1, an item reached consensus for an appropriate proposal if the median score was ≥7 with all individual scores ≥5 or for an inappropriate proposal if the median score was ≤3.5 with all scores ≤5. All other items were judged as uncertain and resubmitted for further scoring. Items with missing data were also resubmitted. For rounds 2 and 3, scoring followed the HAS 2010 recommendations (Fig. 2). From round 2 onward, expert comments were anonymized and included in the review committee’s evaluation. In the event of unclear or divergent results, the review committee decided whether an item should be resubmitted based on its previous scoring patterns and clinical relevance.

## 3 Results

### 3.1 Literature review

Thirty-three articles were included in the literature review. These studies described major complications of neurogenic bladder, such as renal insufficiency, UTIs, hydronephrosis, and VUR. Reported risk factors included clinical, urodynamic, and therapeutic parameters, as well as comorbidities. Literature reviews, systematic reviews, urodynamic studies, and prognostic or predictive scores in both adult and child populations were included. The review informed the development of the proposals for round 1. All selected articles from the review were provided to the rating committee.

### 3.2 Round 1

**Fig. 3:**
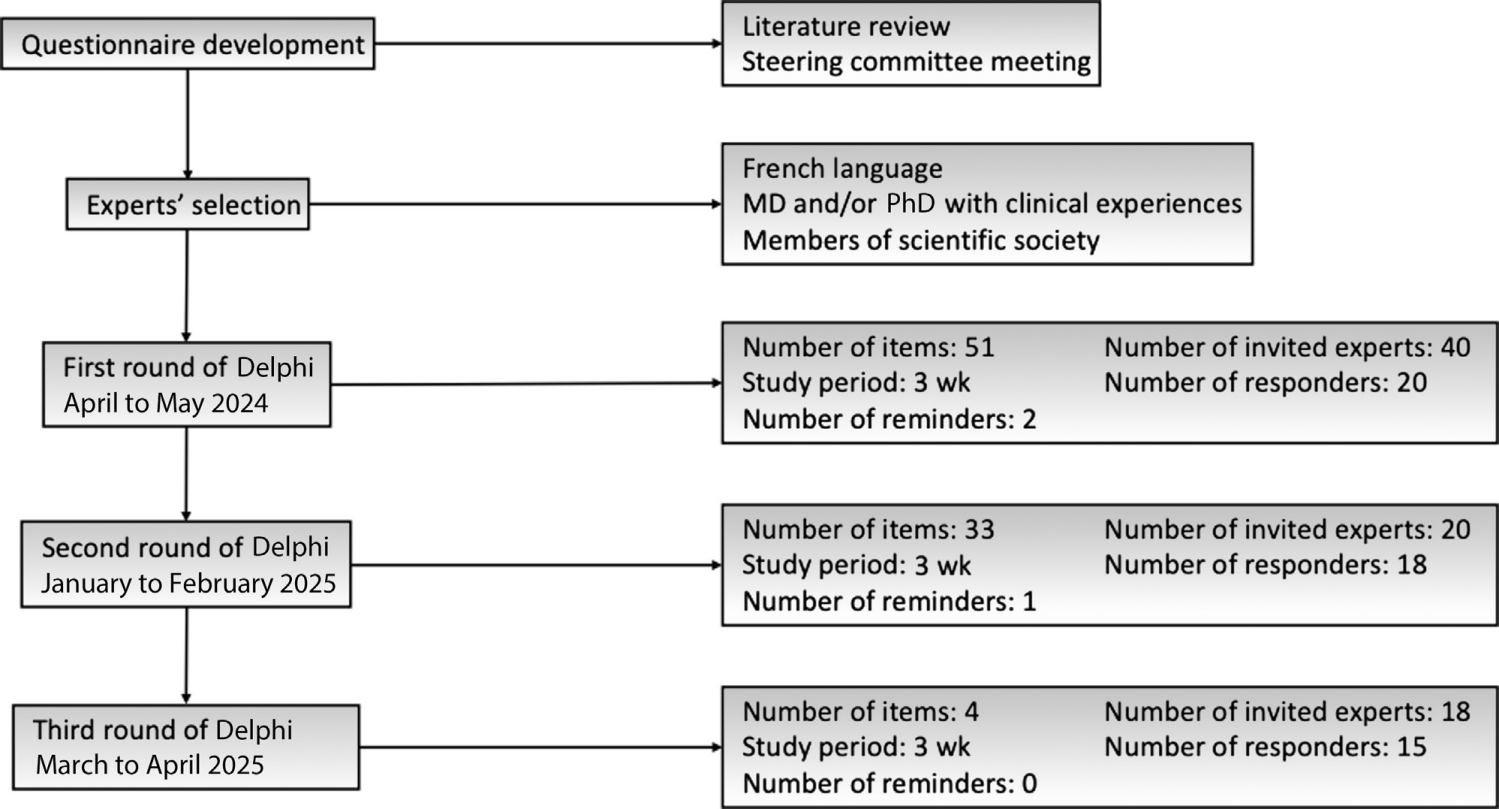
Delphi method flowchart.

Fifty-one items were generated: 11 items addressing complications and 40 items addressing risk factors. Of the 40 invited experts, 20 responded (50%; Fig. 3). None of the 11 complication-related items (Table 2) met the predefined thresholds for agreement or disagreement. The review committee revised and consolidated items to limit redundancy, resulting in six items retained for round 2.

**Table 2:** Complications

Complications	Items	Consensus (round)	Median (min–max)
Kidney	A reduction in renal function (eg, worsening stage of renal failure) should be considered as a warning sign of a complication related to neurogenic bladder	Appropriate Strong agreement (R2)	8 (6–9)
	A glomerular filtration rate of <60 ml/min/1.73 m^2^ should be considered a warning sign of a complication related to neurogenic bladder	Appropriate Strong agreement (R2)	8 (6–9)
	The presence of renal failure should prompt careful evaluation and management of neurogenic bladder	Appropriate Strong agreement (R2)	9 (7–9)
Infection	A urinary tract infection associated with fever should be considered a warning sign of a complication related to neurogenic bladder	Appropriate Strong agreement (R2)	8 (6–9)
Specific (SCI only)	Autonomic dysreflexia should be considered a warning sign of a complication related to neurogenic bladder	Appropriate Strong agreement (R2)	8 (5–9)
Other	The recent onset of hydronephrosis should be recognized as a risk factor for complications in neurogenic bladder	Appropriate Strong agreement (R3)	8 (7–9)

R = round; SCI = spinal cord injury.

In the risk factor section, nine items reached agreement in round 1 (Table 3), including those related to myelomeningocele, SCI, and long-term use of indwelling catheters. The remaining items were revised for clarity. Some were rephrased as “should warn of neurogenic bladder complications” to reflect the need for heightened clinical vigilance rather than certainty of causality. After revision, 28 items were retained for round 2.

**Table 3:** Risk factors of complications

Risk factors	Items	Consensus	Median (min–max)
Clinical	Spinal cord injury is a risk factor for upper urinary tract complications	Appropriate Strong agreement (R1)	9 (8–9)
	Myelomeningocele is a risk of upper urinary tract complications	Appropriate Strong agreement (R1)	9 (7–9)
	Inflammatory spinal cord disease (transverse myelitis, multiple sclerosis, etc.) is a risk of upper urinary tract complications	Appropriate Strong agreement (R1)	8 (5–9)
Treatment	Long-term use of an indwelling urethral catheter is a risk factor for upper urinary tract complications	Appropriate Strong agreement (R1)	9 (7–9)
	Absence of specific bladder management in patients with spinal cord injury is a risk factor for upper urinary tract complications	Appropriate Strong agreement (R1)	9 (8–9)
	Absence of specific bladder management in patients with myelomeningocele is a risk factor for upper urinary tract complications	Appropriate Strong agreement (R1)	9 (8–9)
	Absence of specific bladder management in patients with inflammatory spinal cord disease is a risk factor for upper urinary tract complications	Appropriate Strong agreement (R1)	9 (7–9)
	Long-term use of a suprapubic catheter is a risk factor for complications associated with neurogenic bladder	Appropriate Relative agreement (R2)	9 (5–9)
	Prolonged use of triggered reflex voiding is a risk factor for complications associated with neurogenic bladder	Appropriate Relative agreement (R2)	9 (5–9)
Urodynamics	Bladder compliance <20 ml/cmH_2_O is a risk factor for complications associated with neurogenic bladder	Appropriate Strong agreement (R2)	8 (5–9)
	A detrusor leak point pressure of ≥40 cmH_2_O is a risk factor for complications associated with neurogenic bladder	Appropriate Relative agreement (R2)	8 (5–9)
	The presence of sphincter dyssynergia is a risk factor for complications associated with neurogenic bladder	Appropriate Strong agreement (R2)	8 (5–9)
	The combination of detrusor overactivity and sphincter dyssynergia is a risk factor for complications associated with neurogenic bladder	Appropriate Strong agreement (R2)	8 (5–9)
	A maximum detrusor pressure ≥75 cmH_2_O is a risk factor for complications associated with neurogenic bladder	Appropriate Strong agreement (R1)	9 (5–9)
	Prolonged duration of involuntary detrusor contractions is a risk factor for complications associated with neurogenic bladder	Appropriate Strong agreement (R1)	9 (5–9)
	Presence of poorly controlled diabetes, in addition to a neurological disorder causing bladder dysfunction, is a risk factor for complications associated with neurogenic bladder	Appropriate Relative agreement (R3)	8 (5–9)

R = round.

### 3.3 Round 2

Among the 20 experts who participated in round 1, 18 (90%) participated in round 2. Five items reached strong agreement in the complications section (Table 2), identifying renal failure, febrile UTIs, and autonomic dysreflexia (AD) as the key complications. In the risk factor section, three items reached strong agreement and three achieved relative agreement. No items were rejected. All others were considered uncertain (Table 4). Four items were carried forward to round 3 due to their clinical importance despite uncertainty.

**Table 4:** Propositions with uncertainty or no consensus

Complications	Items	Consensus	Median (min–max)
Other	Presence of ultrasound abnormalities (eg, bladder wall thickening, deformity, and diverticula) is a risk factor for complications associated with neurogenic bladder	Uncertain	7 (1–9)
	Vesicoureteral reflux is a risk factor for complications associated with neurogenic bladder	No consensus	8 (3–9)
Urodynamics	Bladder capacity <200 ml is a risk factor for complications associated with neurogenic bladder	Uncertain	8 (1–9)
	In the case of spontaneous voiding, the presence of a postvoid residual volume is a risk factor for complications associated with neurogenic bladder	No consensus	8 (1–9)
	Absence of bladder sensation during urodynamic evaluation is a risk factor for complications associated with neurogenic bladder	Uncertain	8 (1–9)
	Detrusor overactivity observed during urodynamics is a risk factor for complications associated with neurogenic bladder	Uncertain	8 (1–9)
	A contraction duration to cystometry duration ratio of >0.33 is a risk factor for complications associated with neurogenic bladder	Uncertain	8 (3–9)
Treatment	Lack of specific bladder management in patients with stroke or Parkinson's disease is a risk factor for complications associated with neurogenic bladder	Uncertain	6.5 (1–9)
Clinical	A history of febrile urinary tract infection within the past 12 mo is a risk factor for complications associated with neurogenic bladder	Uncertain	8 (1–9)
	In patients with spinal cord injury, absence of pinprick sensation in sacral dermatomes is a risk factor for complications associated with neurogenic bladder	Uncertain	7.5 (1–9)
	In patients with spinal cord injury, absence of the bulbocavernosus reflex is a risk factor for complications associated with neurogenic bladder	Uncertain	8 (1–9)
	Patients with cerebral lesions (eg, stroke and Parkinson’s disease) are at risk of complications associated with neurogenic bladder	Uncertain	8 (1–9)
	Presence of comorbidities such as hypertension, in addition to a neurological disorder causing bladder dysfunction, is a risk factor for complications associated with neurogenic bladder	Uncertain	8 (1–9)
	A history of kidney stones is a risk factor for complications associated with neurogenic bladder	Uncertain	6.5 (1–9)

### 3.4 Round 3

Fifteen of the 18 experts (83%) participated in round 3. Two additional items achieved strong agreement: poorly controlled diabetes as a risk factor and recent onset of hydronephrosis as a complication (Table 2).

After three Delphi rounds, six items were retained as complications (Table 2) and 16 as risk factors (Table 3), and 14 remained uncertain (Table 4).

## 4 Discussion

Our expert consensus, using the Delphi method, resulted in consensus on 22 proposals, including six related to complications and 16 linked to risk factors, and determined clinical, urodynamic, and management recommendations (Table 5). These recommendations should support practitioners (urologists, neurologists, physiatrists, and others) in clearly identifying high-risk situations and patient groups to facilitate appropriate referral to specialized departments. Identification of high-risk groups is a key component of neurogenic bladder management, helping determine appropriate timing for assessment and follow-up. However, previous studies had not enabled a clear and unified definition for complications or associated risk factors.

**Table 5:** Accepted proposals and experts’ agreement

		Agreement
*Complications*	*Considered a warning sign of a complication related to neurogenic bladder*	
	A reduction in renal function (eg, worsening stage of renal failure)	Strong
	A glomerular filtration rate of <60 ml/min/1.73 m^2^	Strong
	Presence of renal failure should prompt careful evaluation and management of neurogenic bladder	Strong
	A urinary tract infection associated with fever	Strong
	Autonomic dysreflexia	Strong
	Recent onset of hydronephrosis	Strong
*Risk factors*	*For upper urinary tract complications*	
Management	Long-term use of an indwelling urethral catheter	Strong
	Long-term use of a suprapubic catheter	Relative
	Prolonged use of triggered reflex voiding	Relative
Clinical	Absence of specific bladder management in patients with spinal cord injury	Strong
	Absence of specific bladder management in patients with myelomeningocele	Strong
	Absence of specific bladder management in patients with inflammatory spinal cord disease	Strong
Urodynamic	Bladder compliance <20 ml/cmH_2_O	Strong
	Detrusor leak point pressure of ≥40 cmH_2_O	Relative
	Maximum detrusor pressure of ≥75 cmH_2_O	Strong
	Presence of sphincter dyssynergia	Strong
	Prolonged duration of involuntary detrusor contractions	Strong
	Combination of detrusor overactivity and sphincter dyssynergia	Strong
Other	Presence of poorly controlled diabetes, in addition to a neurological disorder causing bladder dysfunction	Relative

The first section of our consensus focused on complications of neurogenic bladder. As highlighted in systematic reviews [6], [7], complications are often defined by morphological abnormalities (hydronephrosis and renal scarring), but few studies refer to renal impairment or renal failure, a life-threatening condition. Our consensus identified six complications, with three related to renal function: renal failure, a decrease in renal function, and glomerular filtration rate <60 ml/min/1.73 m^2^. Febrile UTIs and AD also achieved strong agreement. AD is associated with major cardiovascular events, and its occurrence must be considered systematically, especially in patients with an SCI above T6. The last complication—recent onset of hydronephrosis—reached agreement after three rounds. This proposal generated debate and was ultimately accepted, whereas VUR failed to reach consensus, largely due to diagnostic challenges and the limited clinical value of systematically investigating VUR in the absence of symptoms such as fever or recurrent febrile UTIs. Indeed, even as videourodynamics is considered the gold standard in the assessment of bladder function in patients with neurogenic bladder, the low availability of this test makes regular and systematic VUR detection difficult. For recommendations to be considered useful, these should align with the availability of local resources [12]. On the contrary, hydronephrosis, which is strongly associated with VUR and often considered an endpoint of UUTD (particularly in pediatric cohorts [7]), can be detected via renal ultrasound, a routine and easily accessible imaging test [13], [14].

Despite the heterogeneity present in the definitions of UUTD, numerous studies, reviews, and predictive scores have attempted to identify clinical, urodynamic, and management-related risk factors for UUTD. The second round of our study addressed these risk factors, leading to consensus on 16 proposals.

The primary finding for clinical risk factors was the identification of high-risk groups: patients with myelomeningocele, SCI, and, to a lesser degree, inflammatory spinal cord disease (transverse myelitis and MS). This aligns with the existing literature identifying populations with these conditions as high-risk groups for complications such as renal failure and UTIs [3], [8], [15], [16], [17]. Despite reaching strong agreement on inflammatory spinal cord disease, existing data indicate that MS carries a lower risk than SCI or myelomeningocele. Indeed, renal failure is rare in MS [18], [19], as are VUR and hydronephrosis [16], [20]. In MS, clinical risk factors remain poorly defined and controversial; variables such as the number of lower urinary tract symptoms, disease duration, or Expanded Disability Status Scale score of ≥5 may be associated with urodynamic abnormalities relevant to UUTD [21] but are not directly predictive of UUTD themselves.

Other clinical risk factors have been described, including the typology of spinal dysraphism [15], level of SCI [22], and comorbidities such as diabetes [23]. Diabetes is a well-known independent risk factor for infection or renal failure, and must be monitored and managed as effectively as possible, in addition to the management of neurogenic bladder [23]. The steering committee did not include all such specificities to maintain the simplicity and usability of the recommendations in everyday practice. Among proposed comorbidities, only poorly controlled diabetes reached relative agreement after three rounds.

In addition to clinical factors, two urodynamic risk factors reached strong agreement in the first round: a maximum detrusor pressure of ≥75 cmH_2_O and prolonged duration of involuntary detrusor contraction. Although consensus was achieved by the end of the study, experts did not agree on the threshold value, and the proposed contraction duration to cystometry duration ratio of >0.33 remains uncertain. As noted by Musco et al [7], many urodynamic parameters lack consistent thresholds across studies. Despite this lack of homogeneity, the most studied urodynamic parameters were low bladder compliance (6/21 studies), DLPP (5/21 studies), and the maximum mean value of detrusor pressure. All these parameters were mostly studied in adults with SCI (60%) and children with spina bifida (98%).

In the second round, experts subsequently identified four additional urodynamic risk factors. As expected, DLPP ≥40 cmH_2_O and bladder compliance <20 ml/cmH_2_O were also accepted. In round 1, experts had evaluated whether <20 or <12.5 ml/cmH_2_O was more predictive. As both thresholds reached agreement in round 2, the committees selected <20 ml/cmH_2_O based on higher expert ratings and greater utility in early management.

Low bladder compliance is a well-established risk factor for UUTD and must be assessed and managed promptly, particularly in high-risk groups. DLPP, first described as a urodynamic risk factor for UUTD in 1981 by McGuire et al [4], has since been reassessed with varied thresholds. Galloway et al [5] suggested a threshold of ≥50 cmH_2_O; subsequent studies debated these values [7], [24] and generally proposed lower thresholds [25]. Recent receiver operating characteristic–based analyses [25] and data from adult patients [9] suggest that DLPP ≥15 cmH_2_O may already indicate an increased UUTD risk and should be considered in early management.

Haudebert et al [9] identified three urodynamic predictors of UUTD: bladder compliance (odds ratio [OR] = 0.18), maximum detrusor pressure (OR = 14.7), and detrusor overactivity (OR = 1.84). In our consensus, isolated detrusor overactivity was not accepted, while the combination of detrusor overactivity and DSD reached strong agreement in round 2. Experts also strongly agreed that isolated DSD is a risk factor for UUTD. Despite recent evidence linking DSD to recurrent UTIs and AD in SCI patients [26], its diagnosis remains challenging. DSD typically requires integration of clinical, radiological, and urodynamic data [27], [28]. Videourodynamics is useful, but urodynamics combined with electromyography and voiding cystourethrogram can be alternatives, although electromyography is not recommended as a standalone tool for DSD diagnosis [29], [30]. Limited access to such investigations further complicates routine diagnosis, and currently, DSD diagnosis is usually based on a compatible urodynamic pattern in the context of spinal lesions.

Other debated topics included postvoid residual (PVR). Despite three reformulations, no consensus was achieved. Variations tested PVR thresholds (≥150 ml or ≥50% of bladder capacity), and the final proposal specified excluding patients performing intermittent self-catheterization “in case of spontaneous voiding.” Persistent disagreement reflects the clinical reality: no validated PVR threshold exists for initiating an intervention. The significance of PVR is highly dependent on the underlying etiology. In MS, PVR ≥100 ml may justify intermittent self-catheterization if symptomatic [31], but other studies found no link between PVR and symptom severity [32]. May et al [33] reported no threshold predictive of UTIs. In cauda equina syndrome, PVR ≥200 ml may support diagnosis [34], although further evidence is required. More broadly, this debate highlights the challenge of managing PVR in neurogenic bladder dysfunction.

The final component of our Delphi method process addressed management strategies, and experts achieved consensus on six proposals. Four reached strong agreement in round 1, which emphasized the need for early management of neurogenic bladder, particularly in high-risk groups (SCI, spinal dysraphism, and inflammatory spinal cord diseases). Long-term use of indwelling urethral catheters also reached strong agreement as a known risk factor for UUTD, especially UTIs and AD [26], and should be avoided whenever possible. Two additional proposals achieved relative agreement after round 2, recommending the avoidance of long-term suprapubic catheterization and triggered reflex voiding. These recommendations align with the current guidelines [35], [36], [37], which support intermittent catheterization as the preferred management strategy for patients with voiding dysfunction and significant PVR [38].

The main limitation of this study is the heterogeneity and uncertainty surrounding definitions of UUTD and associated risk factors. This variability justified the use of a Delphi methodology, as many points remain controversial and require prospective studies to establish reliable thresholds. Such studies will be difficult to conduct without large multicenter cohorts or artificial intelligence–driven databases integrating all existing complications and risk factors.

The strengths of our study include the high level of expertise represented and the practical applicability of the recommendations. The steering committee aimed to deliver clear and pragmatic guidance to help neurologists, urologists, and physiatrists detect both high- and low-risk patients early and propose the appropriate referrals. Early identification of risk factors is increasingly recognized as essential in everyday practice and must remain accessible to both expert and nonexpert practitioners. The complex conditions of these patients—who often experience neurological, gastrointestinal, and sexual comorbidities—require clear neurourological risk stratification and structured referral pathways. Our consensus aligns with the prioritization of neurourological risk situations published by Hentzen et al [39] in 2022, which also emphasized the urgency of referral depending on patients’ clinical presentation.

Further prospective multicenter studies will be necessary to statistically validate the predictive scores for risk factors and potential complications of neurogenic bladder. These studies will require large sample sizes and long-term follow-up. The main challenge will be to determine the relative weight of each clinical or urodynamic variable for the different etiologies of neurogenic bladder.

## 5 Conclusions

Our Delphi method expert consensus validated 22 proposals concerning neurogenic bladder—six regarding complications alone and 16 concerning risk factors for complications. This consensus should support practitioners in improving the management of neurogenic bladder patients. These proposals should be integrated within the existing prioritization of situations with important neurourological risks to ensure their comprehensive management and avoid underestimating serious renal or infectious complications. Ideally, future long-term prospective cohort studies will help validate and refine the conclusions of this consensus.

  ***Author contributions*:** Nicolas Turmel had full access to all the data in the study and takes responsibility for the integrity of the data and the accuracy of the data analysis.

  *Study concept and design*: Turmel, Amarenco, Denys, Game, Hentzen.

*Acquisition of data*: Turmel, Hentzen.

*Analysis and interpretation of data*: Turmel, Hentzen, Chesnel.

*Drafting of the manuscript*: Turmel, Hentzen, Amarenco.

*Critical revision of the manuscript for important intellectual content*: Turmel, Amarenco, Denys, Game, Hentzen, Chesnel.

*Statistical analysis*: Turmel, Hentzen, Amarenco.

*Obtaining funding*: Amarenco, Denys, Game.

*Administrative, technical, or material support*: None.

*Supervision*: Hentzen, Amarenco.

*Other*: None.

  ***Financial disclosures:*** Nicolas Turmel certifies that all conflicts of interest, including specific financial interests and relationships and affiliations relevant to the subject matter or materials discussed in the manuscript (eg, employment/affiliation, grants or funding, consultancies, honoraria, stock ownership or options, expert testimony, royalties, or patents filed, received, or pending), are the following: None.

  ***Funding/Support and role of the sponsor*:** This work is part of a project funded by Abbvie. This funding project aims to structure neuro-urology departments nationwide.
